# Magnetocardiography Combined With the SYNTAX Score for Exploratory Modeling of Clinician‐Selected Revascularization Category in Three‐Vessel Coronary Artery Disease: A Single‐Center Pilot Study

**DOI:** 10.1155/crp/9497660

**Published:** 2026-06-12

**Authors:** Ziyu An, Shipan Wang, Min Zhang, Mingduo Zhang, Feng Xu, Lanxin Feng, Zhao Ma, Huan Zhang, Shuwen Yang, Chenchen Tu, Xiantao Song, Hongjia Zhang

**Affiliations:** ^1^ Department of Cardiology, Beijing Anzhen Hospital, Capital Medical University, Beijing, 100029, China, ccmu.edu.cn; ^2^ Department of Cardiovascular Surgery, Beijing Anzhen Hospital, Capital Medical University, Beijing, 100029, China, ccmu.edu.cn; ^3^ Department of Cardiology, Beijing Friendship Hospital, Capital Medical University, Beijing, 100050, China, ccmu.edu.cn

**Keywords:** magnetocardiography, revascularization, SYNTAX score, three-vessel coronary artery disease

## Abstract

**Background:**

Three‐vessel coronary artery disease (3V‐CAD) often requires revascularization with either percutaneous coronary intervention (PCI) or coronary artery bypass grafting (CABG). The SYNTAX score is widely used for anatomical risk stratification, but whether magnetocardiography (MCG) provides incremental information beyond anatomical assessment remains uncertain.

**Objectives:**

To evaluate, in an exploratory pilot analysis, whether MCG‐derived parameters add information beyond the SYNTAX score for modeling clinician‐selected revascularization category in 3V‐CAD and to examine the noncausal association between model‐treatment concordance and major adverse cardiovascular and cerebrovascular events (MACCEs).

**Methods:**

Prospective cohort data were analyzed retrospectively. Candidate MCG parameters were screened using Pearson correlation, random forest analysis, and stepwise logistic regression, and selected variables were combined with the SYNTAX score. Model discrimination, calibration, bootstrap internal validation, and LASSO penalized logistic regression sensitivity analysis were assessed. Model‐treatment concordance was explored using Kaplan–Meier analysis and multivariable Cox regression.

**Results:**

Among 544 patients, 543 complete cases were available for model evaluation, including 42 CABG events. In the overall cohort, the combined MCG‐SYNTAX model did not materially improve discrimination compared with the SYNTAX‐only model (AUC, 0.853 vs. 0.847; *p* = 0.628). Discrimination was also similar in the low‐risk subgroup (SYNTAX < 22; AUC, 0.824 vs. 0.848; *p* = 0.540). In the intermediate‐high‐risk subgroup (SYNTAX ≥ 22), the combined model had a numerically higher apparent AUC, but this finding was considered hypothesis‐generating. Bootstrap internal validation did not support a maintained incremental value of the combined model (optimism‐corrected AUC, 0.834 vs. 0.848 for the SYNTAX‐only model). In LASSO sensitivity analysis using all appended MCG‐derived candidate variables, only the SYNTAX score was retained. The exploratory follow‐up analysis showed an unadjusted difference in MACCE‐free survival between concordance groups, but this association was not retained after multivariable adjustment and was not interpreted causally.

**Conclusions:**

In this single‐center pilot modeling study, selected MCG parameters showed limited and unstable incremental value beyond the SYNTAX score for modeling clinician‐selected revascularization category in 3V‐CAD. These findings do not support clinical implementation at this stage and require external validation.

**Trial Registration:** Chinese Clinical Trial Registry: ChiCTR2200066942

## 1. Introduction

Three‐vessel coronary artery disease (3V‐CAD) is characterized by obstructive atherosclerotic lesions, defined as luminal stenosis of ≥ 50%, involving the left anterior descending artery, left circumflex artery, and right coronary artery [1]. Severe three‐vessel disease may cause myocardial ischemia and present as stable or unstable angina; in advanced cases, it may progress to myocardial infarction, heart failure, or sudden cardiac death [2]. In addition to medical therapy, many patients require revascularization with either percutaneous coronary intervention (PCI) or coronary artery bypass grafting (CABG) [3].

The European System for Cardiac Operative Risk Evaluation (EuroSCORE) was one of the earliest tools used to inform operative risk assessment, but it may overestimate mortality risk [4]. The SYNTAX score, based on angiographic lesion complexity, provides more specific anatomical information for revascularization planning in 3V‐CAD [5]. Although PCI is generally considered acceptable in patients with a SYNTAX score ≤ 22, the relative benefit of PCI versus CABG remains debated in patients with intermediate SYNTAX scores (23–32).

Magnetocardiography (MCG) is a noninvasive imaging technique that records and reconstructs magnetic fields generated by cardiac electrical activity. Previous studies from our group suggested that MCG may have diagnostic value for coronary artery disease [6], with reported sensitivity and specificity of 95.1% and 92.8%, respectively [7]. MCG has also shown good agreement with myocardial perfusion imaging in assessing myocardial ischemia [8]. Therefore, MCG‐derived functional information may theoretically complement the anatomically based SYNTAX score in evaluating revascularization category.

We therefore conducted a single‐center pilot modeling study to explore whether selected MCG‐derived parameters add information beyond the SYNTAX score for modeling clinician‐selected revascularization category in three‐vessel disease. We also performed an exploratory follow‐up analysis to examine the noncausal association between model‐treatment concordance and subsequent major adverse cardiovascular and cerebrovascular event (MACCE).

## 2. Methods

### 2.1. Design and Study Population

**FIGURE 1:**
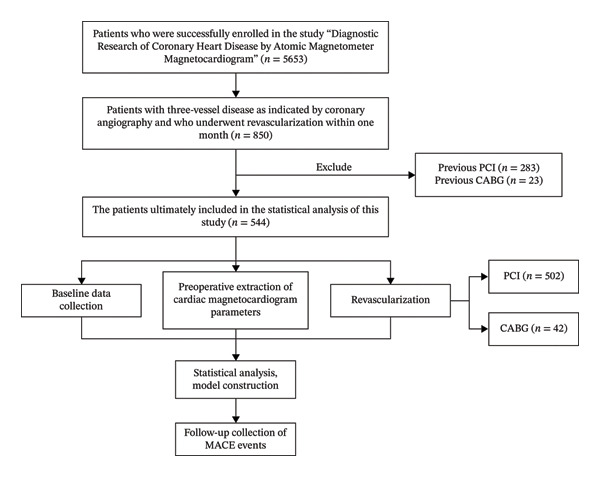
Flowchart of patient selection. Abbreviations: PCI, percutaneous coronary intervention; CABG, coronary artery bypass grafting; MACCE, major adverse cardiovascular and cerebrovascular event.

This single‐center study was conducted at the Coronary Heart Disease Center of Beijing Anzhen Hospital, Capital Medical University. Data were derived from the prospective registered study “Diagnosis of Coronary Heart Disease by Atomic Magnetometer Cardiography” and analyzed retrospectively. Participants enrolled between December 21, 2022, and December 13, 2023, were screened. Patients were eligible if they had ≥ 50% stenosis in all three major epicardial coronary arteries and were scheduled for coronary revascularization within 1 month. Patients with previous PCI or CABG were excluded. Among 850 screened patients with 3V‐CAD, 544 met the eligibility criteria (Figure 1).

The model‐development outcome was clinician‐selected revascularization strategy (PCI or CABG) after coronary angiography. This outcome was used as a real‐world reference for treatment selection, not as a gold standard for optimal revascularization. Treatment decisions were made by cardiologists alone or jointly by cardiologists and cardiac surgeons, all blinded to the MCG results.

### 2.2. MCG System and Recording

All MCG recordings were performed using a 36‐channel optically pumped magnetometer system (Miracle MCG, Beijing X‐MAGTECH Technologies Ltd., Beijing, China), with a measurement sensitivity of < 30 fT/Hz^1/2^. The residual direct‐current magnetic field in the measurement area was controlled to < 5 nT, and the system was operated without additional room shielding. During acquisition, the patient lay supine, and the 6 × 6 sensor array was positioned approximately 2 cm above the chest. Cardiac magnetic field signals were recorded continuously from 36 channels for 90 s. Potential external magnetic interference was removed according to the institutional MCG protocol.

### 2.3. MCG Data Analysis

**FIGURE 2:**
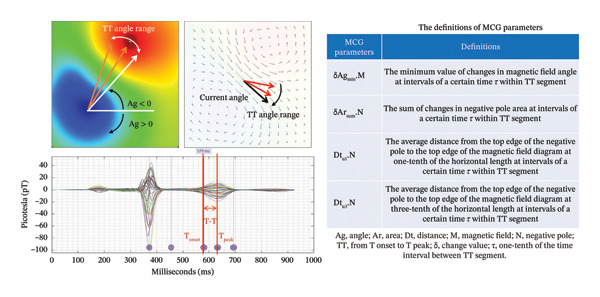
Schematic illustration of MCG current‐angle parameters. In the magnetic‐field distribution map (left) and current‐density map (right), angle values are defined relative to the horizontal axis; counterclockwise angles are negative and clockwise angles are positive. The TT segment is defined as the interval from T‐wave onset to T‐wave peak. Abbreviation: MCG, magnetocardiography.

The system software reconstructed magnetic‐field maps and pseudo‐current density maps, which displayed the spatiotemporal distribution of cardiac magnetic activity and generated quantitative parameter outputs. Image quality and parameter outputs were reviewed by an independent investigator, and poor‐quality recordings were excluded. A total of 65 MCG‐derived parameters related to magnetic‐field amplitude, current‐angle dynamics, waveform timing, magnetic pole changes, and spatiotemporal signal characteristics were collected. The definition of MCG current‐angle parameters is illustrated in Figure 2. The naming convention and plain‐language interpretation of the retained MCG variables are summarized in Supporting Table S6.

### 2.4. ICA

Invasive coronary angiography was performed during hospitalization according to standard institutional protocols. Stenosis severity and revascularization strategy were determined by experienced interventional cardiologists. Three‐vessel disease was defined as ≥ 50% stenosis in all three major epicardial coronary arteries.

### 2.5. SYNTAX Score

All patients with three‐vessel coronary lesions included in the study underwent coronary angiography, and the SYNTAX score was calculated using the SYNTAX score calculator (Version 2.28). Two experienced interventional cardiologists independently calculated the SYNTAX score. When their assessments were inconsistent, a third interventional cardiologist participated in the adjudication. Patients were stratified into two prespecified SYNTAX risk groups: low risk (SYNTAX score < 22) and intermediate‐high risk (SYNTAX score ≥ 22).

### 2.6. Exploratory Follow‐Up Analysis

The exploratory follow‐up endpoint was MACCE, defined as a composite of all‐cause death, unplanned revascularization, myocardial infarction, and stroke. Follow‐up duration was calculated from revascularization to the first MACCE event, last clinical follow‐up, or loss to follow‐up.

Patients were classified as consistent or inconsistent according to whether the actual treatment matched the treatment category predicted by the combined MCG‐SYNTAX model. This analysis was descriptive and exploratory and was not intended to infer a causal effect of model‐treatment concordance on clinical outcomes.

### 2.7. Statistical Analysis

Continuous variables are presented as mean (SD), and categorical variables are presented as *n* (%). Group comparisons were performed using Student’s *t*‐test or the chi‐square/Fisher exact test, as appropriate. Logistic regression was used to construct the primary prediction models. Discrimination was assessed using receiver operating characteristic (ROC) analysis and area under the curve (AUC), with AUCs compared using the DeLong test. ROC curves were smoothed for visualization only; AUC values and DeLong *p* values were calculated from the original empirical ROC curves.

Complete‐case analysis was used for model evaluation. One patient was excluded because of missing data in variables required for model assessment, leaving 543 patients in the complete‐case model‐evaluation cohort. Calibration was assessed using calibration plots, Brier scores, and Hosmer–Lemeshow testing. Internal validation was performed using bootstrap resampling.

As a sensitivity analysis, penalized logistic regression using the least absolute shrinkage and selection operator (LASSO) was performed to assess variable‐selection robustness in the low‐event setting. The SYNTAX score was forced into the model without penalization, whereas all appended MCG‐derived candidate variables were penalized. This analysis was exploratory and was not intended to replace the primary logistic regression model.

Threshold probability, sensitivity, and specificity were derived using the Youden index. Kaplan–Meier analysis and the log‐rank test were used for unadjusted survival comparisons. Multivariable Cox proportional hazards regression was used to examine the adjusted association between model‐treatment concordance and MACCE. A two‐sided *p* value < 0.05 was considered statistically significant.

## 3. Results

### 3.1. Study Cohort and Model Construction

A total of 544 patients were included in the baseline cohort (mean age, 60.91 ± 9.60 years; 403 men, 74.1%), including 42 patients (7.7%) who underwent CABG (Table 1). One patient had missing data required for model evaluation and was excluded from discrimination, calibration, and internal validation analyses, leaving 543 patients in the complete‐case model‐evaluation cohort.

**TABLE 1:** Clinical characteristics by SYNTAX risk groups.

Characteristic	Overall	Low risk (< 22)	Intermediate‐high risk (≥ 22)	*p* value
*n*	544	421	123	
Age, years	60.91 (9.60)	60.54 (9.71)	62.15 (9.18)	0.102
Male sex, *n* (%)	403 (74.1)	308 (73.2)	95 (77.2)	0.429
BMI, kg/m^2^	25.98 (3.34)	25.99 (3.37)	25.95 (3.24)	0.905
Smoking, *n* (%)	120 (22.1)	90 (21.4)	30 (24.4)	0.558
Hypertension, *n* (%)	352 (64.7)	271 (64.4)	81 (65.9)	0.845
Diabetes mellitus, *n* (%)	219 (40.3)	167 (39.7)	52 (42.3)	0.678
Chronic kidney disease, *n* (%)	15 (2.8)	12 (2.9)	3 (2.4)	1.000
Peripheral arterial disease, *n* (%)	20 (3.7)	12 (2.9)	8 (6.5)	0.105
LVEF, %	62.53 (6.26)	62.97 (5.99)	61.13 (6.90)	**0.005**
STEMI, *n* (%)	7 (1.3)	5 (1.2)	2 (1.6)	1.000
NSTEMI, *n* (%)	33 (6.1)	27 (6.4)	6 (4.9)	0.680
Unstable angina, *n* (%)	499 (91.7)	385 (91.4)	114 (92.7)	0.802
Stable angina, *n* (%)	3 (0.6)	2 (0.5)	1 (0.8)	1.000
δAgmin.M	−8.84 (28.29)	−7.96 (26.76)	−11.88 (32.92)	0.176
δArsum.N	16115.62 (15285.19)	16019.45 (14799.35)	16444.77 (16903.51)	0.786
Dtu1.N	139.93 (127.47)	143.68 (129.35)	127.13 (120.46)	0.206
Dtu3.N	218.07 (114.05)	223.53 (112.23)	199.45 (118.66)	0.039
CABG, *n* (%)	42 (7.7)	14 (3.3)	28 (22.8)	**< 0.001**
SYNTAX score	16.97 (6.90)	14.03 (3.98)	27.01 (5.09)	**< 0.001**

*Note:* Data are presented as *n* (%) or mean (SD). Significant *p* values are shown in bold. Grouping was defined as low risk (SYNTAX < 22) and intermediate‐high risk (SYNTAX ≥ 22).

Given the limited number of CABG events, the final number of MCG variables was restricted. Among 65 candidate MCG parameters, Pearson correlation and random forest screening identified five candidate variables, and stepwise logistic regression retained four variables: δAgmin.M, δArsum.N, Dtu1.N, and Dtu3.N (Supporting Figure S1 and Supporting Table S1). δAgmin.M and δArsum.N represent summary measures of magnetic‐field/current‐angle change, whereas Dtu1.N and Dtu3.N reflect timing‐related features from the reconstructed MCG waveform. The naming convention and plain‐language descriptions of these variables are provided in Supporting Table S6.

In the complete‐case cohort, the apparent AUC was 0.853 for the combined model and 0.847 for the SYNTAX‐only model (Table 2). Bootstrap internal validation yielded optimism‐corrected AUCs of 0.834 and 0.848, respectively (Supporting Table S3), indicating that the apparent incremental value of the combined model was not maintained.

**TABLE 2:** Discriminative performance of the combined MCG‐SYNTAX model versus the SYNTAX‐only model.

Subgroup	N	CABG events	Combined model AUC	SYNTAX‐only AUC	DeLong *p* value
Overall cohort	543	42	0.853	0.847	0.628
Low risk (< 22)	420	14	0.824	0.848	0.540
Intermediate‐high risk (≥ 22)	123	28	0.714	0.615	**0.003**

*Note:* AUC, area under the receiver operating characteristic curve; MCG, magnetocardiography. Significant *p* values are shown in bold.

Abbreviation: CABG, coronary artery bypass grafting.

**FIGURE 3:**
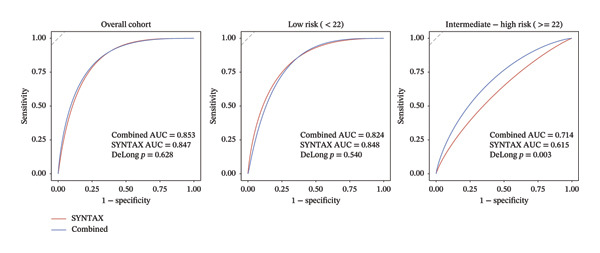
Receiver operating characteristic (ROC) analysis comparing the combined MCG‐SYNTAX model with the SYNTAX‐only model. (Left) Overall cohort. (Middle) Low‐risk subgroup (SYNTAX < 22). (Right) Intermediate‐high‐risk subgroup (SYNTAX ≥ 22). ROC curves were smoothed for visualization only; area under the curve (AUC) values and DeLong test *p* values were calculated from the original empirical ROC curves.

### 3.2. Exploratory Model Performance Assessment

In the complete‐case cohort, the combined MCG‐SYNTAX model did not materially improve discrimination compared with the SYNTAX‐only model in the overall cohort (AUC, 0.853 vs. 0.847; DeLong *p* = 0.628; Table 2 and Figure 3 (Left)). In the low‐risk subgroup (SYNTAX < 22), discrimination was similar between models (AUC, 0.824 vs. 0.848; *p* = 0.540; Figure 3 (middle)). In the intermediate‐high‐risk subgroup (SYNTAX ≥ 22), the apparent AUC was numerically higher for the combined model (AUC, 0.714 vs. 0.615; *p* = 0.003; Figure 3 (right)). However, given the limited event count, data‐driven modeling, and lack of maintained overall advantage after bootstrap validation, this subgroup finding should be interpreted strictly as hypothesis‐generating.

**FIGURE 4:**
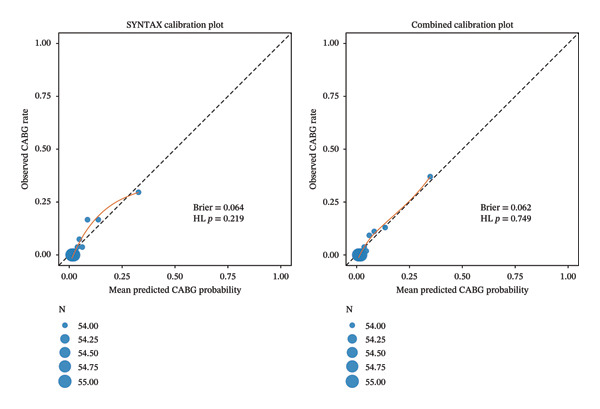
Calibration plots for the SYNTAX‐only model and the combined MCG‐SYNTAX model in the complete‐case model‐evaluation cohort. Points represent grouped observed event rates, the dashed line indicates ideal calibration, and the smoothed line shows the observed calibration trend.

Calibration was acceptable for both models (Figure 4 and Supporting Table S4). Threshold probability, sensitivity, and specificity are provided in Supporting Table S2. Decision‐curve analysis showed minimal separation between models and did not provide strong evidence of clinically meaningful net benefit (Supporting Figure S4). In the LASSO sensitivity analysis using all appended MCG‐derived candidate variables, only the SYNTAX score was retained under both the λmin and *λ*1se rules. The LASSO combined model did not improve cross‐validated discrimination compared with the SYNTAX‐only model (cross‐validated AUC, 0.833 vs. 0.843; Supporting Table S7).

### 3.3. Exploratory Follow‐Up Analysis According to Model‐Treatment Concordance

Among the 543 patients included in the exploratory follow‐up analysis, 385 were classified as consistent and 158 as inconsistent according to concordance between the combined model‐predicted treatment category and actual treatment (Supporting Table S5). Median follow‐up was 636 days. During follow‐up, 37 patients experienced MACCE, and 1 patient was lost to follow‐up (0.18%).

**FIGURE 5:**
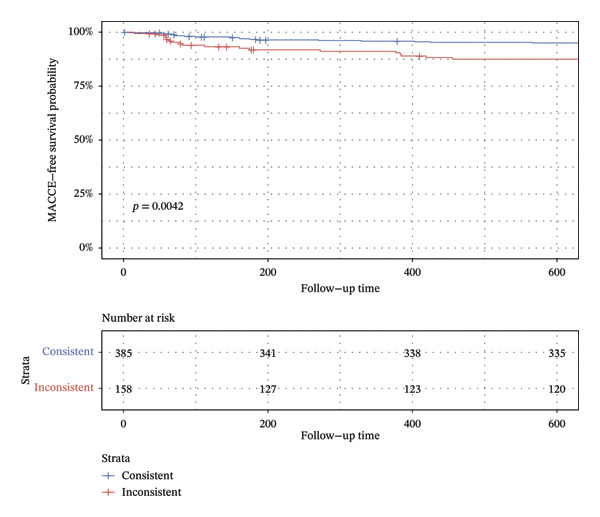
Unadjusted Kaplan–Meier curves for MACCE‐free survival according to concordance between the combined model‐predicted treatment category and the actual treatment strategy. MACCE was defined as a composite of all‐cause death, unplanned revascularization, myocardial infarction, and stroke.

Baseline characteristics differed between groups, particularly in LVEF, SYNTAX score, stable angina, and CABG frequency (Supporting Table S5). Unadjusted Kaplan–Meier analysis showed a difference in MACCE‐free survival between groups (Figure 5). However, model‐treatment inconsistency was not independently associated with MACCE after multivariable adjustment (HR, 1.084; 95% CI, 0.416–2.825; *p* = 0.869), whereas SYNTAX score (HR, 1.098; *p* = 0.002) and CABG versus PCI (HR, 0.079; *p* = 0.037) remained associated with outcome (Table 3). Given baseline imbalance and treatment‐selection bias, this analysis should be interpreted only as descriptive and does not support a causal prognostic interpretation of model‐treatment concordance.

**TABLE 3:** Multivariable Cox regression for MACCE.

Variable	Hazard ratio	95% CI	Z statistic	*p* value
Inconsistent vs. consistent	1.084	0.416–2.825	0.165	0.869
Age, per 1 year	1.006	0.970–1.044	0.343	0.731
Male sex	0.997	0.463–2.150	−0.007	0.995
SYNTAX score, per 1 point	1.098	1.035–1.165	3.096	**0.002**
CABG vs. PCI	0.079	0.007–0.858	−2.086	0.037
LVEF, per 1%	1.044	0.982–1.109	1.373	0.170
Diabetes mellitus	1.735	0.884–3.404	1.602	0.109
Peripheral arterial disease	3.050	0.858–10.842	1.723	0.085

*Note:* Significant p values are shown in bold.

Abbreviations: CABG, coronary artery bypass grafting; LVEF, left ventricular ejection fraction; MACCE, major adverse cardiovascular and cerebrovascular event; PCI, percutaneous coronary intervention.

## 4. Discussion

In this single‐center pilot modeling study, adding selected MCG parameters to the SYNTAX score did not materially improve discrimination for modeling clinician‐selected revascularization category in the overall cohort. Although the apparent AUC was numerically higher in the intermediate‐high‐risk subgroup, this finding should be interpreted strictly as hypothesis‐generating because of the limited number of CABG events, the data‐driven modeling process, and the clinician‐selected endpoint.

The overall evidence did not support a robust incremental contribution of MCG‐derived variables beyond the SYNTAX score. Bootstrap internal validation attenuated the apparent performance of the combined model, and LASSO sensitivity analysis retained only the SYNTAX score when all appended MCG‐derived candidate variables were penalized. Decision‐curve analysis also showed minimal separation between models. In addition, although unadjusted MACCE‐free survival differed between concordance groups, this association was not retained after multivariable adjustment. Together, these findings support interpreting the model as a methodological pilot rather than a clinically actionable decision‐support tool.

Revascularization decision‐making in 3V‐CAD remains complex. Medical therapy is fundamental, but revascularization may be important in patients with extensive disease or impaired ventricular function [9]. Previous randomized trials, meta‐analyses, and registry studies have shown that the relative benefit of PCI versus CABG depends on anatomical complexity, clinical risk profile, and long‐term endpoints rather than a uniform treatment effect [10–14]. The SYNTAX trial and its long‐term follow‐up further highlight this uncertainty, particularly among patients outside the low‐risk range, where anatomical assessment alone may not fully resolve treatment selection [1, 15, 16]. This uncertainty provided the rationale for exploring whether MCG‐derived functional information could complement the SYNTAX score.

Functional assessment has been used to refine treatment selection in complex coronary disease, as illustrated by FFR‐guided PCI studies [17]. MCG is noninvasive, noncontact, and radiation‐free, and it records the electromagnetic activity of the heart. Because MCG is sensitive to tangential currents and circular eddy currents generated during myocardial ischemia, it may capture subtle ischemia‐related electrophysiological abnormalities [18, 19].

Several studies have supported the diagnostic potential of MCG in myocardial ischemia. Park et al. reported favorable diagnostic performance of MCG for detecting coronary artery disease in patients with acute chest pain [7]. Our previous work also showed that an MCG‐based machine learning model could predict myocardial perfusion abnormalities verified by single‐photon emission computed tomography, suggesting that MCG may reflect perfusion‐related functional impairment [8]. In addition, our group developed an MCG‐based diagnostic model for myocardial ischemia in borderline coronary lesions, with an AUC of 0.864, suggesting potential value for identifying ischemic burden before invasive angiography [20]. However, the present study differs from these diagnostic studies because it modeled clinician‐selected revascularization category rather than ischemia or perfusion abnormalities. In this setting, MCG‐derived variables did not show a stable incremental role beyond the SYNTAX score. The retained variables remain technical signal‐derived features, and their reproducibility, biological interpretation, and external validity require further study.

### 4.1. Limitations

This study has several limitations. First, the model‐development outcome was clinician‐selected revascularization strategy rather than an independently adjudicated optimal‐treatment standard; therefore, residual selection bias, circularity, and confounding by indication cannot be excluded. Second, the number of CABG events was limited, and the modeling workflow was data‐driven rather than prespecified. Although bootstrap validation and LASSO sensitivity analysis were added, the optimism‐corrected AUCs did not support a clear incremental advantage over the SYNTAX‐only model, and no MCG‐derived variable was retained after penalization. These findings support interpreting the apparent MCG contribution as unstable and hypothesis‐generating. Third, although a glossary was added to improve interpretability of the retained MCG variables, reproducibility across repeated recordings, operators, or independent datasets was not formally assessed, and biological interpretation remains indirect. Fourth, this was a single‐center study without external validation, limiting generalizability. Finally, the follow‐up analysis was descriptive and exploratory; model‐treatment concordance was not independently associated with MACCE after multivariable adjustment and should not be interpreted causally.

## 5. Conclusion

In this single‐center pilot modeling study, selected MCG parameters showed limited and unstable incremental value beyond the SYNTAX score for modeling clinician‐selected revascularization category in 3V‐CAD. Bootstrap validation and LASSO sensitivity analysis did not support a maintained advantage of the combined model, and the subgroup finding should be considered hypothesis‐generating. The follow‐up analysis was exploratory and does not justify causal interpretation or clinical implementation. External validation and reproducibility studies are required before any clinical application can be considered.

## Author Contributions

Xiantao Song and Chenchen Tu contributed to the conception of the topic. Ziyu An and Shipan Wang wrote the manuscript and conducted the statistical analysis. Zhao Ma, Lanxin Feng, and Huan Zhang performed the data collection and collation. Min Zhang and Mingduo Zhang helped to finish the statistics. Shuwen Yang and Feng Xu helped to structure the text. Hongjia Zhang and Chenchen Tu helped to revise the manuscript.

## Funding

This study was supported by Capital’s Funds for Health Improvement and Research, 2024‐2‐2066; Coordinated Innovation of Scientific and Technological in Beijing‐Tianjin‐Hebei Region, Z231100003923008; Beijing Nova Program, 20220484222; Beijing Hospitals Authority Clinical Medicine Development of Special Funding, ZLRK202317; High‐Level Public Health Technical Talent Construction Project of Beijing Municipal Health Commission, Leading Talent‐02‐01; Project of the Beijing Lab for Cardiovascular Precision Medicine, PXM2018_014226_000013; and Beijing Municipal Science & Technology Commission, Z221100007422015.

## Disclosure

All authors have reviewed the final version of the manuscript and approved it for publication. The funders had no role in study design, data collection and analysis, manuscript writing, or the decision to submit the article for publication.

## Ethics Statement

This study used data from a prospective, single‐center, observational cohort, and the present analysis was performed retrospectively. The study protocol has been reviewed by the Ethics Committee of Beijing Anzhen Hospital, Capital Medical University, and has been registered in the “Chinese Clinical Trial Registry.” All participants signed the informed consent form. The methods described in this article follow the diagnostic accuracy reporting standards (STARD) 2015 guidelines.

## Conflicts of Interest

The authors declare no conflicts of interest.

## Supporting Information

Additional supporting information can be found online in the Supporting Information section.

## Supporting information


**Supporting Information** The supporting materials include seven supporting tables and four supporting figures. Supporting Table S1 summarizes the AUCs of different MCG combination models evaluated during model construction. Supporting Table S2 provides the optimal threshold, sensitivity, and specificity of the SYNTAX‐only model and the combined MCG‐SYNTAX model. Supporting Table S3 presents the bootstrap internal validation results for model discrimination, including apparent AUC, mean optimism, and optimism‐corrected AUC. Supporting Table S4 summarizes calibration metrics, including the Brier score and Hosmer–Lemeshow test results. Supporting Table S5 presents baseline characteristics according to model‐treatment concordance. Supporting Table S6 provides the naming convention for MCG‐derived variables and plain‐language descriptions of the retained MCG variables. Supporting Table S7 presents the LASSO penalized logistic regression sensitivity analysis using all appended MCG‐derived candidate variables. Supporting Figure S1 shows the variable‐screening process for MCG model construction, including random‐forest variable‐importance ranking and correlation analysis. Supporting Figure S2 illustrates the relationship between SYNTAX score and the predicted probability of CABG from the combined MCG‐SYNTAX model. Supporting Figure S3 shows the distributions of predicted CABG probabilities generated by the combined MCG‐SYNTAX model and the SYNTAX‐only model according to actual treatment category. Supporting Figure S4 presents the decision‐curve analysis comparing the combined MCG‐SYNTAX model with the SYNTAX‐only model in the overall cohort and prespecified SYNTAX subgroups.

## Data Availability

The datasets used or analyzed during the current study are available from the corresponding author on reasonable request.
