# Supplementary material for: Magnetocardiography Combined With the SYNTAX Score for Exploratory Modeling of Clinician‐Selected Revascularization Category in Three‐Vessel Coronary Artery Disease: A Single‐Center Pilot Study
Source: Cardiol Res Pract. 2026 Jun 12;2026:9497660. doi: 10.1155/crp/9497660 (PMC13263640; doi:10.1155/crp/9497660)

**Supplementary Table S1 The AUC of different MCG combination models**

| **Variables** | | | | **AUC** |
| --- | --- | --- | --- | --- |
| Dtu1.N | Dtu3.N | RtoAm.RpTp | RtoAm.RpTpN | 0.8285 |
| Dtu1.N | Dtu3.N | RtoAm.RpTp | δAgmin.M | 0.8294 |
| Dtu1.N | Dtu3.N | RtoAm.RpTp | δArsum.N | 0.8286 |
| Dtu1.N | Dtu3.N | RtoAm.RpTpN | δAgmin.M | 0.8333 |
| Dtu1.N | Dtu3.N | RtoAm.RpTpN | δArsum.N | 0.8332 |
| Dtu1.N | Dtu3.N | δAgmin.M | δArsum.N | 0.8337 |
| Dtu1.N | RtoAm.RpTp | RtoAm.RpTpN | δAgmin.M | 0.8268 |
| Dtu1.N | RtoAm.RpTp | RtoAm.RpTpN | δArsum.N | 0.8223 |
| Dtu1.N | RtoAm.RpTp | δAgmin.M | δArsum.N | 0.8231 |
| Dtu1.N | RtoAm.RpTpN | δAgmin.M | δArsum.N | 0.8321 |
| Dtu3.N | RtoAm.RpTp | RtoAm.RpTpN | δAgmin.M | 0.8196 |
| Dtu3.N | RtoAm.RpTp | RtoAm.RpTpN | δArsum.N | 0.8265 |
| Dtu3.N | RtoAm.RpTp | δAgmin.M | δArsum.N | 0.824 |
| Dtu3.N | RtoAm.RpTpN | δAgmin.M | δArsum.N | 0.8302 |
| RtoAm.RpTp | RtoAm.RpTpN | δAgmin.M | δArsum.N | 0.8224 |

**Supplementary Table S2. Threshold, sensitivity, and specificity of the two models**

| **Model** | **Optimal threshold** | **Sensitivity** | **Specificity** |
| --- | --- | --- | --- |
| Syntax | 0.067 | 0.833 | 0.731 |
| Combined | 0.059 | 0.881 | 0.695 |

*Thresholds were normalized from the exported CSV because the source file contained duplicated column names for the two models. Values themselves were preserved.*

**Supplementary Table S3. Bootstrap internal validation of model discrimination**

| **Model** | **Apparent AUC** | **Mean optimism** | **Optimism-corrected AUC** | **Bootstrap / usable iterations** |
| --- | --- | --- | --- | --- |
| Syntax | 0.847 | -0.001 | 0.848 | 500 / 500 |
| Combined | 0.853 | 0.019 | 0.834 | 500 / 500 |

**Supplementary Table S4. Calibration metrics of the two models**

| **Model** | **Brier score** | **Hosmer-Lemeshow P value** |
| --- | --- | --- |
| Syntax | 0.064 | 0.219 |
| Combined | 0.062 | 0.749 |

**Supplementary Table S5. Baseline characteristics by model-treatment concordance**

| **Characteristic** | **Overall** | **Consistent** | **Inconsistent** | **P value** |
| --- | --- | --- | --- | --- |
| n | 543 | 385 | 158 |  |
| Age, years | 60.90 (9.61) | 60.65 (9.74) | 61.51 (9.29) | 0.342 |
| Male sex, n (%) | 402 (74.0) | 288 (74.8) | 114 (72.2) | 0.594 |
| BMI, kg/m² | 25.98 (3.35) | 25.87 (3.30) | 26.25 (3.45) | 0.239 |
| Hypertension, n (%) | 351 (64.6) | 250 (64.9) | 101 (63.9) | 0.901 |
| Diabetes mellitus, n (%) | 219 (40.3) | 159 (41.3) | 60 (38.0) | 0.535 |
| Smoking, n (%) | 120 (22.1) | 86 (22.3) | 34 (21.5) | 0.924 |
| Chronic kidney disease, n (%) | 15 (2.8) | 9 (2.3) | 6 (3.8) | 0.513 |
| LVEF, % | 62.52 (6.26) | 63.13 (5.92) | 61.11 (6.82) | **0.001** |
| Peripheral arterial disease, n (%) | 20 (3.7) | 16 (4.2) | 4 (2.5) | 0.508 |
| STEMI, n (%) | 7 (1.3) | 6 (1.6) | 1 (0.6) | 0.653 |
| NSTEMI, n (%) | 33 (6.1) | 21 (5.5) | 12 (7.6) | 0.453 |
| Unstable angina, n (%) | 498 (91.7) | 356 (92.5) | 142 (89.9) | 0.410 |
| Stable angina, n (%) | 3 (0.6) | 0 (0.0) | 3 (1.9) | 0.038 |
| SYNTAX score | 16.97 (6.91) | 14.45 (5.68) | 23.10 (5.69) | **<0.001** |
| CABG, n (%) | 42 (7.7) | 37 (9.6) | 5 (3.2) | 0.017 |

*Data are presented as n (%) or mean (SD). Significant P values are shown in bold. Concordance refers to agreement between the actual treatment and the treatment category predicted by the combined model using the selected decision threshold.*

**Supplementary Table S6. MCG variables and naming convention used in this study**

**Panel A. Naming convention used for MCG-derived variables**

| Symbol / abbreviation | Meaning |
| --- | --- |
| Dt | Distance |
| Ps | Position |
| Ag | Angle |
| Am | Amplitude |
| Ar | Area |
| Rto | Ratio |
| P | Positive pole |
| PP | Positive pole point |
| N | Negative pole |
| NP | Negative pole point |
| M | Magnetic field |
| C | Current |
| max | Maximum |
| min | Minimum |
| sum | Cumulative sum |
| std | Standard deviation |
| δ | Temporal change |
| u | Upper boundary |
| d | Lower boundary |
| TT segment | Interval from T-wave onset to T-wave peak |

**Panel B. Plain-language description of retained MCG variables in the final model**

| **Variable** | **Functional domain** | **Plain-language description** |
| --- | --- | --- |
| Dtu1-N | Spatial boundary-distance feature | Average distance from the upper boundary of the negative pole to the top edge of the magnetic-field map at the 1/10 horizontal position during the TT segment |
| Dtu3-N | Spatial boundary-distance feature | Average distance from the upper boundary of the negative pole to the top edge of the magnetic-field map at the 3/10 horizontal position during the TT segment |
| δAgmin-M | Magnetic-field angle dynamics feature | Minimum temporal change in magnetic-field angle during the TT segment |
| δArsum-N | Negative-pole area dynamics feature | Cumulative temporal change in negative-pole area during the TT segment |

The retained variables in the final model mainly represent three functional domains during ventricular repolarization: spatial boundary-distance features of the negative magnetic pole, magnetic-field angle dynamics, and negative-pole area dynamics. The TT segment was defined as the interval from T-wave onset to T-wave peak.

**Supplementary Table S7. LASSO penalized logistic regression sensitivity analysis using all appended MCG-derived candidate variables**

This table summarizes an exploratory penalized logistic regression sensitivity analysis performed to assess whether MCG-derived variables provided robust incremental information beyond the SYNTAX score under LASSO regularization.

| **Model** | **Candidate predictors** | **Selected predictors** | **Selected MCG variables, n** | **Apparent AUC** | **Cross-validated AUC** | **P vs. SYNTAX-only model** | **N** | **CABG events, n** |
| --- | --- | --- | --- | --- | --- | --- | --- | --- |
| SYNTAX-only logistic regression | SYNTAX score | SYNTAX score | 0 | 0.847 | 0.843 | — | 543 | 42 |
| LASSO combined model (lambda.min) | SYNTAX score forced in + 86 penalized MCG-derived variables | SYNTAX score | 0 | 0.847 | 0.833 | 0.013 | 543 | 42 |
| LASSO combined model (lambda.1se) | SYNTAX score forced in + 86 penalized MCG-derived variables | SYNTAX score | 0 | 0.847 | 0.833 | 0.013 | 543 | 42 |

Note: In the LASSO combined models, the SYNTAX score was forced into the model and was not penalized, whereas all appended MCG-derived variables were penalized.

The lambda.min model corresponds to the value of the regularization parameter that maximized cross-validated AUC. The lambda.1se model corresponds to the most regularized model within one standard error of the maximum cross-validated AUC.

This analysis was performed as an exploratory sensitivity analysis and should not be interpreted as external validation.

Abbreviations: AUC, area under the curve; CABG, coronary artery bypass grafting; LASSO, least absolute shrinkage and selection operator; MCG, magnetocardiography; SYNTAX, Synergy Between Percutaneous Coronary Intervention With Taxus and Cardiac Surgery.

**Supplementary Figure S1**


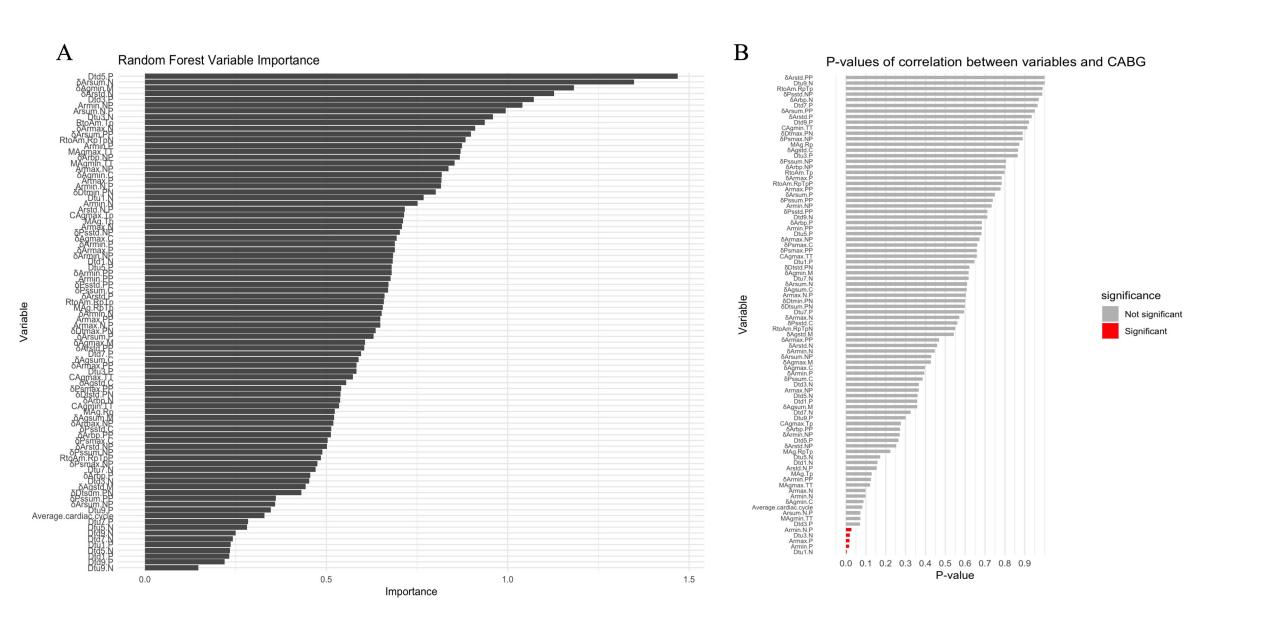


**Supplementary Figure S2**

**
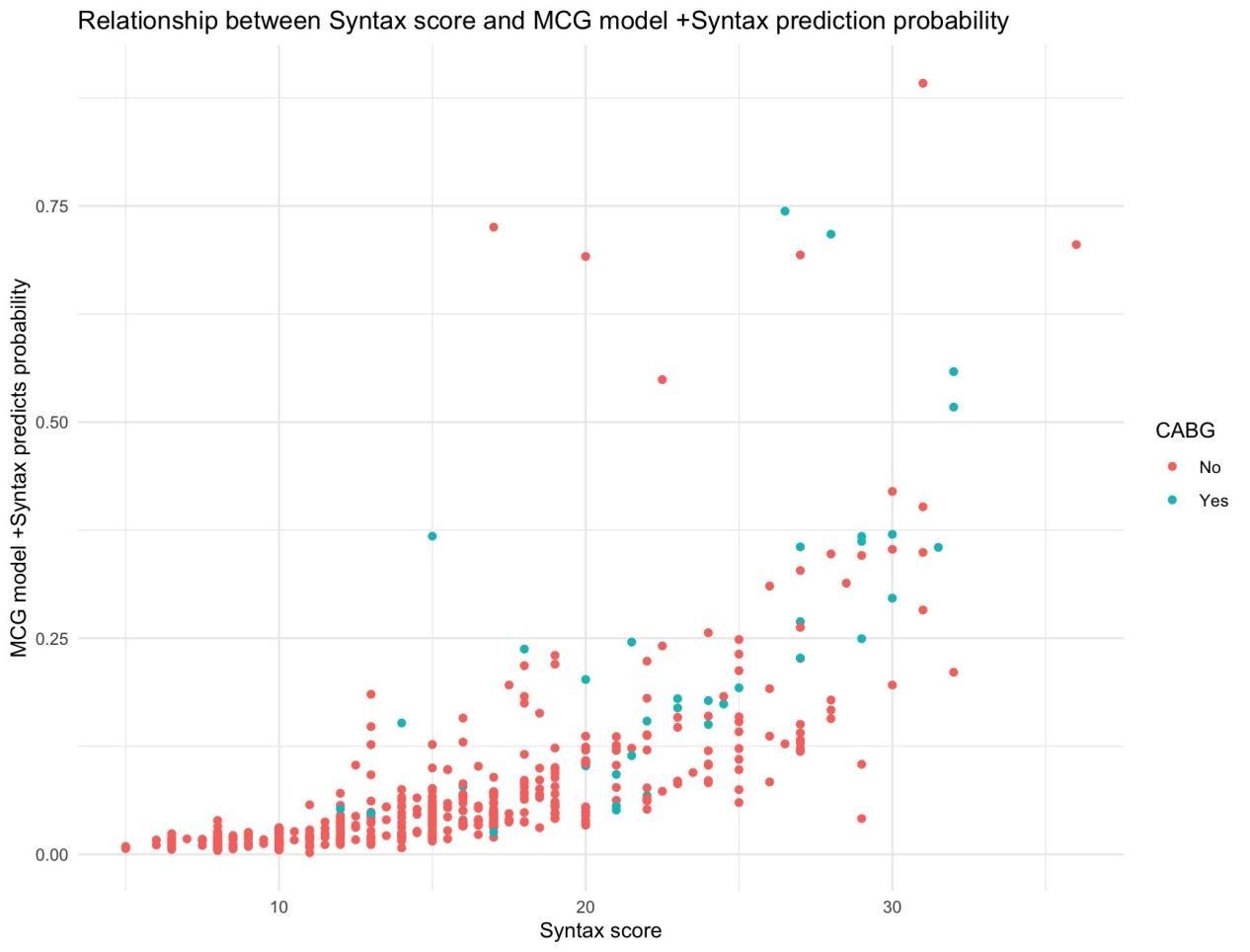
**

**Supplementary Figure S3**


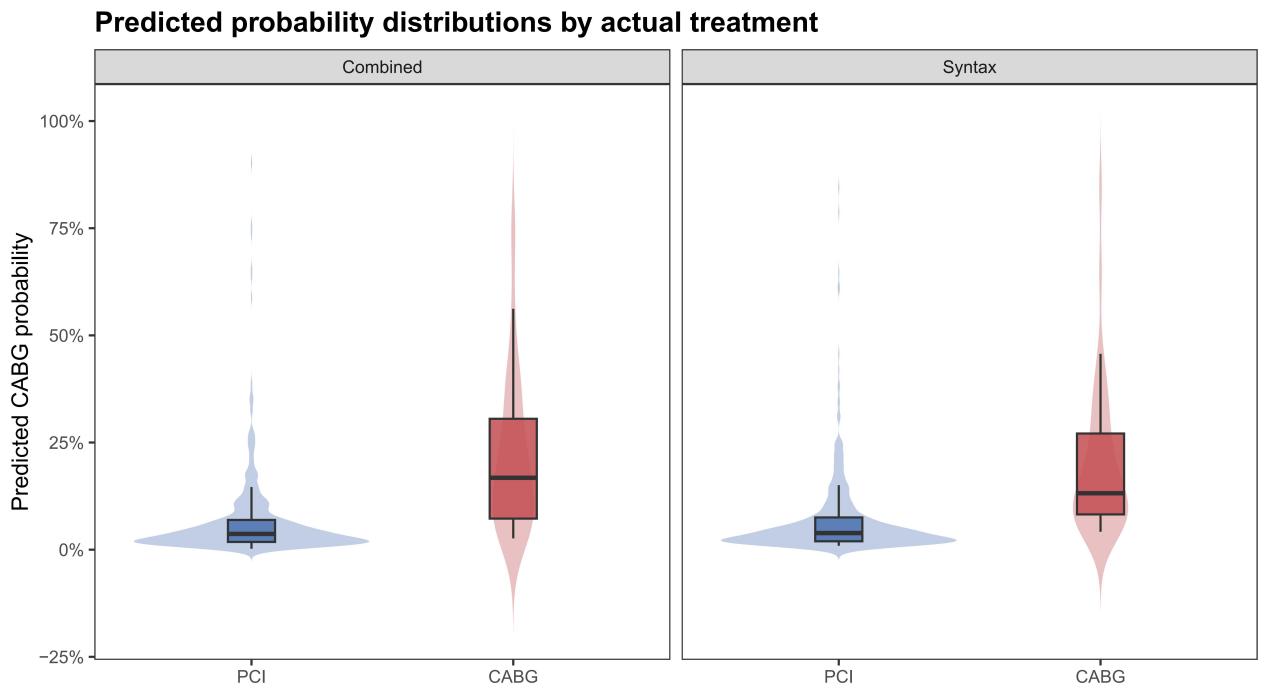


**Supplementary Figure S4**


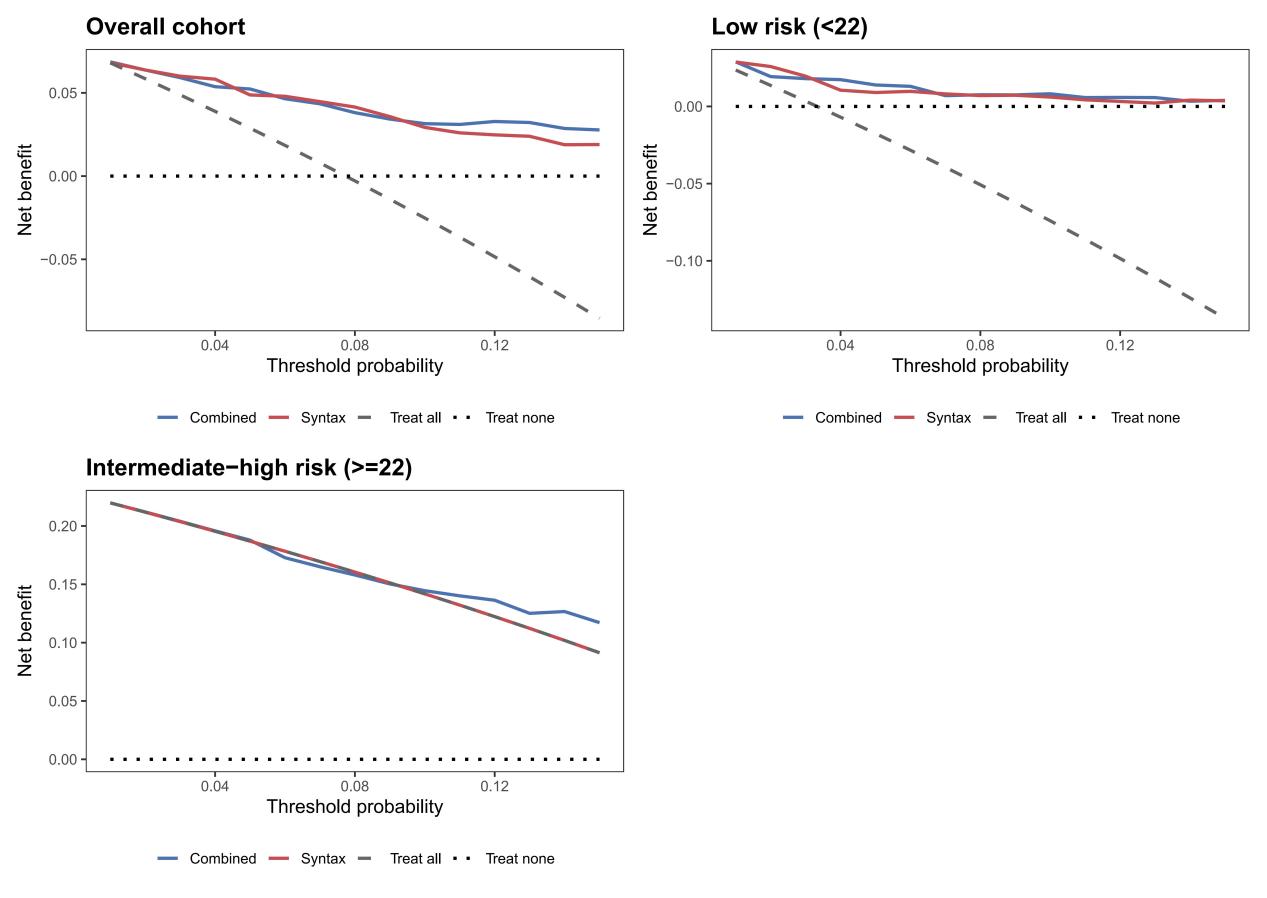

Supplement: Supplementary file 1 — Supporting Information The supporting materials include seven supporting tables and four supporting figures. Supporting Table S1 summarizes the AUCs of different MCG combination models evaluated during model construction. Supporting Table S2 provides the optimal threshold, sensitivity, and specificity of the SYNTAX‐only model and the combined MCG‐SYNTAX model. Supporting Table S3 presents the bootstrap internal validation results for model discrimination, including apparent AUC, mean optimism, and optimism‐corrected AUC. Supporting Table S4 summarizes calibration metrics, including the Brier score and Hosmer–Lemeshow test results. Supporting Table S5 presents baseline characteristics according to model‐treatment concordance. Supporting Table S6 provides the naming convention for MCG‐derived variables and plain‐language descriptions of the retained MCG variables. Supporting Table S7 presents the LASSO penalized logistic regression sensitivity analysis using all appended MCG‐derived candidate variables. Supporting Figure S1 shows the variable‐screening process for MCG model construction, including random‐forest variable‐importance ranking and correlation analysis. Supporting Figure S2 illustrates the relationship between SYNTAX score and the predicted probability of CABG from the combined MCG‐SYNTAX model. Supporting Figure S3 shows the distributions of predicted CABG probabilities generated by the combined MCG‐SYNTAX model and the SYNTAX‐only model according to actual treatment category. Supporting Figure S4 presents the decision‐curve analysis comparing the combined MCG‐SYNTAX model with the SYNTAX‐only model in the overall cohort and prespecified SYNTAX subgroups. [file CRP-2026-9497660-s001.docx]
